# Pulmonary function in adults with recent and former asthma and the role of sex and atopy

**DOI:** 10.1186/1471-2466-12-32

**Published:** 2012-06-29

**Authors:** Yue Chen, Donna C Rennie, Punam Pahwa, James A Dosman

**Affiliations:** 1 Department of Epidemiology and Community Medicine, Faculty of Medicine, University of Ottawa, 451, Smyth Road, Ottawa, Ontario, K1H 8 M5, Canada; 2 Canadian Centre for Health and Safety in Agriculture, University of Saskatchewan, 103, Hospital Drive, P.O. Box 120, R.U.H, Saskatoon, Saskatchewan, S7N 0 W8, Canada; 3 College of Nursing, University of Saskatchewan, 107, Wiggins Road, Saskatoon, Saskatchewan, S7N 5E5, Canada

**Keywords:** Asthma, Atopy, Survey, Lung function, Sex

## Abstract

**Background:**

Pulmonary function is not fully reversible in asthma in children and may continue into adult life. This study was to determine the association between asthma and reduced pulmonary function in adults and the modification by sex and atopic status.

**Methods:**

A cross-sectional study of 1492 adults aged 18 years or over was conducted in a rural community. Atopy, height, weight, waist circumference (WC) and pulmonary function were measured. Participants with ever asthma were those who reported by questionnaire a history of asthma diagnosed by a physician during lifetime. Participants who had former (only) asthma were those who reported having physician-diagnosed asthma more than 12 months ago. Participants who had recent asthma were those who reported having asthma during the last 12 months.

**Results:**

Men had higher values of forced vital capacity (FVC) and forced expiratory volume in one second (FEV_1_) compared with women, but FEV_1_/FVC ratio showed no significant difference between sexes. Atopic status was not related to pulmonary function and the average values of the pulmonary function testing variables were almost the same for non-atopic and atopic individuals. Individuals with ever, recent or former asthma had significant lower values of FEV_1_ and FEV_1_/FVC ratio than those who reported having no asthma, and the associations tended to be stronger in men than in women. The interaction between atopy and asthma was not statistically significant.

**Conclusions:**

Adults who reported having recent asthma or former asthma had reduced pulmonary function, which was significantly modified by sex but not by atopic status.

## Background

Asthma is an inflammatory airway disease and airway function may not be completely reversible in those with asthma. Studies have revealed reduced pulmonary function in individuals with clinically stable asthma [1] or a history of asthma [2,3] in children and young adults. Chronic airway inflammation may result in long-term pulmonary function reduction in asthma patients [4,5] including thickening of the airway wall and the development of incompletely reversible airway narrowing, airway hyperresponsiveness, and reduced airway distensibility [6]. The anomalies may occur early in disease pathogenesis [7,8], and continues into adult life [9], however data from adult populations are scarce. Atopy may [10] or may not [1,11] influence the association between asthma and pulmonary function. In this study we examined the association between asthma and pulmonary function and the effect modification of sex and atopy in a general population of adults to clarify these issues.

## Methods

### Study population

In the present analysis we used data from a cross-sectional study conducted in the town of Humboldt, Saskatchewan, in 2003. A total of 2057 adults participated in the study, 71% of the target population of all town residents aged 18 to 79 years. The study was approved by the University of Saskatchewan research ethics board and consent was obtained from each participant. The details have been described in previous reports [12-14].

### Questionnaire

All the participants completed a self-administrated questionnaire, and provided information including demographic factors, education, occupation, income, smoking habits, coffee and alcohol consumption, respiratory symptoms and illnesses. Participants with ever asthma were those who responded positively to one or both of the following question: 1) “During the past 12 months, has a doctor ever said you had asthma?”; and 2) “Before the past 12 months, has a doctor ever said you had asthma?”. Participants with former (only) asthma were those who responded “no” to the first question but “yes” the second question. Participants with recent asthma were those who responded “yes” to the first question.

Individuals who reported smoking every day or almost everyday, and had smoked at least 20 packs during their lifetime, were defined as current smokers. People who were regular smokers but at the time of the survey, had quit for at least 6 months, were considered as former smokers. Otherwise, subjects were defined as non-smokers. Perceived level of physical activities was also recorded. Physical activity in spare time was measured by asking the question: “Compared to the way other people your age now spend their spare time, would you say you are: more physically active, equally physically active or less physically active?” We use the same definitions in previous reports [12-14].

### Clinical measurements

During a clinical visit, lung function, height, weight and waist circumference (WC) were measured. Weight was measured to the nearest 0.1 kg. Height and WC were measured in centimetres. Height was measured using a fixed tape measure with participants standing shoeless on a hard surface. Waist circumference was measured between the lowest rib and the iliac crest, horizontally through the narrowest part of the torso [15]. BMI was calculated as weight (kg)/height^2^ (m^2^).

Skin prick testing included 4 allergens: *D pteronyssinus,* mixed grasses # (Western Allergy, Vancouver, CA)*, Fel d, Alternaria tenius,* as well as positive (histamine) and negative (saline) controls (Omega Laboratories and Hollister-Stier, Mississauga, CA). Adults were considered atopic if they had a raised wheal greater than or equal to 3 mm compared to the saline control on skin prick testing [16].

A MedGraphics CPF-S System (Medical Graphics Corporation. St. Paul, MN 55127, 1992) was used for pulmonary function testing. Two machines were calibrated using a standard syringe every morning during the study period. Each subject was tested based on the American Thoracic Society criteria defined in the 1987 Standardization of Spirometry [17]. Forced vital capacity (FVC), forced expiratory volume in one second (FEV_1_) and FEV_1_/FVC (%) were included in this analysis. Values were corrected to body temperature and pressure saturated with water vapour (BTPS).

### Statistical analysis

We examined the associations of pulmonary function with ever, recent and former asthma and their variations between men and women and between atopic and non-atopic individuals by using multivariate analysis of variance (MANOVA). FVC, FEV_1_ and FEV_1_/FVC(%) were considered simultaneously, and sex, age, height, body weight, WC and pack-years of smoking (one pack-year is equal to 20 cigarettes smoked per day for a year) were adjusted. Adjusted means and 95% confidence intervals for pulmonary function testing variables were calculated for subjects with and without asthma and their differences associated with sex and atopic status were determined and related interactions were tested. Associations were considered to be statistically significant if p values were less than 0.05 (two sides). All the analyses were conducted by using SPSS version 11.5.

## Results

Of 2057 participants, 1492 (72.5%) provided all related questionnaire information, had pulmonary function testing and skin prick testing results and height, weight and WC measures. Table 1 describes the distribution of anthropometric measures, pulmonary function testing variables and pack-years of smoking according to the status of ever asthma stratified by sex and atopic status.

**Table 1:** Anthropometric measures, pulmonary function and smoking by sex and atopy in subjects with and without asthma

	**Sex**		**Atopy**		
	**Men**	**Women**	**No**	**Yes**	**Total**
Subjects without asthma	(n = 674)	(n = 818)	(n = 1060)	(n = 432)	(n = 1492)
Age (years)	51.2 ± 15.2^*^	50.5 ± 15.7	51.7 ± 15.2	48.7 ± 15.8	50.8 ± 15.5
Weight(kg)	90.2 ± 15.9	74.4 ± 16.3	80.7 ± 17.7	83.6 ± 18.2	81.5 ± 17.9
Height (m)	175.5 ± 6.3	162.5 ± 6.3	168.1 ± 8.9	169.1 ± 9.2	168.4 ± 9.0
BMI^*†*^(kg/m^2^)	29.2 ± 4.8	28.2 ± 6.0	28.4 ± 5.5	29.2 ± 5.7	28.7 ± 5.5
WC^*†*^(cm)	100.5 ± 12.1	87.8 ± 14.0	92.9 ± 14.6	95.1 ± 14.3	93.6 ± 14.6
Packyears	9.5 ± 14.5	5.8 ± 15.8	7.9 ± 16.7	6.4 ± 11.3	7.5 ± 15.4
FVC^*†*^(L)	4.77 ± 0.99	3.43 ± 0.76	3.99 ± 1.09	4.14 ± 1.09	4.04 ± 1.10
FEV_1_^*†*^(L)	3.80 ± 0.86	2.78 ± 0.65	3.20 ± 0.91	3.33 ± 0.90	3.24 ± 0.91
FEV_1_/FVC(%)	79.6 ± 7.5	80.9 ± 6.0	80.2 ± 6.7	80.6 ± 6.7	80.3 ± 6.7
Subjects with ever asthma	(n = 33)	(n = 89)	(n = 68)	(n = 54)	(n = 122)
Age (years)	51.2 ± 16.4	47.5 ± 16.4	52.9 ± 16.7	42.9 ± 14.4	48.5 ± 16.4
Weight(kg)	86.5 ± 14.9	78.3 ± 15.5	80.4 ± 16.6	80.6 ± 14.8	80.5 ± 15.7
Height (m)	175.6 ± 7.5	162.6 ± 6.1	165.4 ± 9.2	166.9 ± 8.1	166.1 ± 8.7
BMI(kg/m^2^)	28.1 ± 4.6	29.7 ± 6.3	29.4 ± 5.9	29.1 ± 5.9	29.3 ± 5.9
WC(cm)	99.3 ± 12.1	92.5 ± 14.5	95.5 ± 15.2	92.9 ± 12.7	94.3 ± 14.2
Packyears	9.5 ± 16.6	7.1 ± 22.7	7.3 ± 15.0	8.3 ± 27.1	7.7 ± 21.1
FVC(L)	4.58 ± 1.06	3.41 ± 0.88	3.49 ± 1.00	4.02 ± 1.06	3.72 ± 1.06
FEV_1_(L)	3.46 ± 1.07	2.74 ± 0.82	2.73 ± 0.88	3.20 ± 0.97	2.94 ± 0.95
FEV_1_/FVC(%)	74.8 ± 9.93	79.6 ± 8.1	77.7 ± 9.7	79.0 ± 7.7	78.3 ± 0.89

^*^Mean ± standard deviation.

^*†*^*BMI*, body mass index; *FVC*, forced vital capacity; *FEV*_*1*_, forced expired volume in one second; *WC*, waist circumference.

In multivariate analysis, we first examined the independent associations of FVC and FEV_1_ and FEV_1_/FVC ratio with ever asthma, sex and atopy. Age, height, weight, WC and pack-years of smoking were important predictors for FVC and FEV_1_ and/or FEV_1_/FVC ratio and were included in the regression models (Table 2). Body mass index was no longer significant predictor and was excluded from the models. Interactions for these variables with asthma status were not statistically significant at the alpha level of 0.05. Ever asthma was significantly associated with reduced FEV_1_ and FEV_1_/FVC ratio, while atopy showed no impact on the pulmonary function testing variables. Women had lower mean values of FVC and FEV_1_ compared with men, but average FEV_1_/FVC ratio was almost the same.

**Table 2:** Adjusted means and 95% confidence intervals of lung function testing variables by sex, atopy and asthma (n = 1614)^*^

	**FVC^*†*^(L)**		**FEV_1_^*†*^(L)**		**FEV_1_/FVC(%)**	
	**Mean**	**95% CI**	**Mean**	**95% CI**	**Mean**	**95% CI**
Sex						
Men	4.39	4.34-4.45	3.54	3.49-3.59	80.6	79.9-81.2
Women	3.72	3.67-3.76	2.97	2.93-3.01	79.9	79.3-80.4
P value	<0.001		<0.001		0.158	
Atopy						
No	4.01	3.98-4.04	3.22	3.19-3.24	80.2	79.8-80.6
Yes	4.02	3.97-4.07	3.22	3.18-3.27	80.1	79.6-80.7
P value	0.668		0.776		0.924	
Ever asthma						
No	4.02	3.99-4.05	3.23	3.20-3.26	80.4	80.0-80.7
Yes	3.92	3.82-4.02	3.07	2.98-3.16	77.8	76.7-79.0
P value	0.050		0.001		<0.001	

^*^Multivariate analysis of variance; models included sex, age, height, weight, waist circumference, smoking, atopy and asthma.

^*†*^*FVC*, forced vital capacity; *FEV*_*1*_, forced expired volume in one second; *SE*, standard error; *WC*, waist circumference.

**Figure 1:**
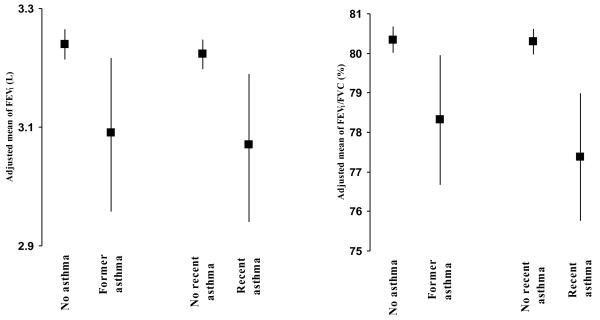
**Adjusted means of FEV**_**1**_**and FEV**_**1**_**/FVC in subjects with former asthma (n = 58) and recent asthma (n = 64) compared with those with no asthma history.**

Similar results were revealed when former asthma and recent asthma were considered separately. Significantly lower values of FEV_1_ and FEV_1_/FVC ratio were observed in those with former asthma than those with no asthma and in those with recent asthma than those without recent asthma (Figure 1).

The association between asthma and reduced FEV_1_ was more pronounced in men than in women and in non-atopic than atopic individuals. Table 3 shows that on average people with ever asthma had a reduction of 0.33 L of FEV_1_ compared to non-asthmatic persons in men (p<0.05), and the corresponding reduction was only 0.09 L for women (p > 0.05). The interaction between asthma and sex was statistically significant. Ever asthma was associated with a reduction of 0.20 L of FEV_1_ in people with no atopy (p<0.05) and with only 0.10 L in atopic people (p > 0.05), but the interaction between ever asthma and atopy was not statistically significant. Similarly, the association between ever asthma and FEV_1_/FVC ratio was statistically significant in men and non-atopic people, but not in women and atopic people, while the interaction between asthma and atopy did not reach statistical significance. We did not detect other important effect modifiers for the association between asthma status and pulmonary function testing variables.

**Table 3:** **Adjusted means and 95% confidence intervals of lung function testing variables related to ever asthma according to sex and atopy (n = 1614)**^***†***^

**Sex/Atopy**	**Ever asthma**	**FVC^*†*^(L)**		**FEV_1_^*†*^(L)**		**FEV_1_/FVC(%)**	
		**Mean**	**95% CI**	**Mean**	**95% CI**	**Mean**	**95% CI**
Sex							
Men	No	4.41	4.35-4.46	3.56	3.51-3.61	80.9	80.2-81.5
	Yes	4.21	4.02-4.41	3.23	3.05-3.40	76.2	74.0-78.5
Women	No	3.72	3.67-3.77	2.97	2.93-3.02	80.0	79.4-80.5
	Yes	3.65	3.54-3.77	2.88	2.78-2.99	78.2	76.9-79.6
P value for asthma x sex		0.283		0.024		0.033	
Atopy							
No	No	4.02	3.99-4.05	3.23	3.20-3.26	80.4	80.0-80.8
	Yes	3.88	3.75-4.02	3.03	2.91-3.15	77.5	75.9-79.1
Yes	No	4.03	3.97-4.08	3.23	3.18-3.28	80.3	79.7-80.9
	Yes	3.96	3.81-4.12	3.12	2.99-3.25	78.2	76.5-79.9
P value for asthma x atopy		0.524		0.346		0.511	

^*^Multivariate analysis of variance; models included sex age, height, weight, waist circumference, smoking, atopy and asthma as well as sex x asthma or atopy x asthma interaction term.

^*†*^*FVC*, forced vital capacity; *FEV*_*1*_, forced expired volume in one second; *SE*, standard error; *WC*, waist circumference.

## Discussion

Our data demonstrated that adults with asthma had reduced airway function indicated by FEV_1_ and FEV_1_/FVC ratio in adults. This reduced airway function was observed not only in adults with recent asthma but also in those with only former asthma. Our results are consistent with earlier observations among children and young adults. Nakadate and Kagawa [3] investigated 441 primary school children and found reduced lung function with a history of asthma even if they had been in remission for several years. Yang et al. [1] found reduced lung function in 242 children with clinically stable asthma compared with 100 non-asthmatic controls. Mosfeldt Laursen et al. [2] studied 77 children or young adults aged 12–24 years with former asthma, current asthma or no asthma and demonstrated that former asthmatics with no current respiratory symptoms had obstructive airflow limitation and increased bronchial responsiveness.

Our data suggest that in adults with recent or former asthma, pulmonary function was not fully reversible. The damage to airway function is likely due to pathological modification of the bronchial airway structures or airway remodeling. Although the mechanisms are not fully understood, there is an expansion of the airway wall vascular compartment involving both enlargement of existing vascular structures and the formation of new vessels [6], and an increased smooth muscle mass and airway narrowing [18]. Damage to the epithelium may play an important role in the process, which can be caused by exposures to pathogens, allergens, environmental pollutants, cigarette smoke, and mechanical injuries [19]. Irreversible loss of pulmonary function in asthma can begin in childhood and continue into adult life [9]. In a cohort from birth to the age of 26 years Rasmussen et al. [9] found that asthma in childhood was associated with a low postbronchodilator ratio of FEV_1_ to vital capacity at the age of 18 and 26 years, and with an accelerated decline in pulmonary function and decreased reversibility. Thus, asthma may cause some permanent airway function damage. Irreversible airway obstruction is related to poor prognosis in asthma [20].

In the present study, we found that sex was a significant effect modifier for the association between asthma and pulmonary function. The reduction in pulmonary function due to recent or former asthma was more marked in men than in women. A previous study showed that asthma and male sex were independently associated with low postbronchodilator ratio of FEV_1_ to vital capacity [9]. In animal studies, sex influences the remodeling process in asthma [21,22], and possible reasons need to be further explored. Reporting bias should not be ignored for the observed sex difference if men tended to under-report and/or women to over-report asthma. In our study, women were more likely to have asthma than men, and this has been observed in previous surveys in the same region and in Canada. A recent report demonstrated that female sex was an independent risk factor for the incidence of non-allergic asthma [23]. Non-allergic asthma was more likely to be under-diagnosed but the diagnoses bias was similar for men and women [23] The differences in asthma prevalence and incidence need to be further explored.

Our data demonstrated that atopy had no notable impact on pulmonary function. Some previous cross-sectional and longitudinal studies have shown no significant associations between atopy and FEV_1_ in adults [24,25]. One study found that atopy alone was not related to the FEV_1_, but in atopic patients the relationship between bronchial hyperresponsiveness and FEV_1_ was stronger compared with nonatopic patients [26]. Other studies demonstrated that atopy was related to lower lung function in young adults with asthma [27] or independently of asthma [28]. Atopy seems not an independent risk factor for pulmonary function in children [1,29], although atopy was found to be associated with low pulmonary function in children with asthma or wheeze [30]. Our study showed that atopy did not significantly modify the association between asthma and pulmonary function and was not an important risk factor for pulmonary function in adults.

Among the study subjects, 27 reported using asthma medication during the past 12 months. Self-reported asthma medication use during the past 12 months did not significantly predict pulmonary function or modified the association between asthma and pulmonary function testing variables. One limitation of the study is that asthma diagnosis is solely based on self-reporting. The current study did not have information on bronchial hyperreactivity. If pulmonary function was fully reversible in asthma and chronic obstructive pulmonary disease (COPD) was misdiagnosed as asthma, we would have observed reduced pulmonary function in “asthma” patients. Since COPD is common in elderly people but rare in young adults, we analyzed the data stratified by age. The association between self-reported asthma and pulmonary function was similar in younger and older age groups. In addition, self-reported former asthma, which is not likely to be COPD, was significantly related to low pulmonary function, suggesting possible information bias is not an explanation for our findings. In this study we used four most important allergens for our skin prick test and 28% were positive. A larger panel of allergens would increase the likelihood of atopy detected, and there was a potential under-diagnosis of atopy in our study.

## Conclusions

There was reduced pulmonary function in adults with ever, recent or former asthma and the association tended to be stronger in men than in women, but similar in those with and without atopy. There is a possibility that airway remodeling in asthma results in reduced pulmonary function and risk factors for irreversible airway obstruction need further investigation.

## Abbreviations

BMI, Body mass index; FEV1, Forced expiratory volume in the first second; BTPS, Body temperature and pressure saturated with water vapour; FVC, Forced vital capacity; FEF, Forced expiratory flow rate; MANOVA, Multivariate analysis of variance; SE, Standard error; WC, Waist circumference.

## Competing interests

The authors declare that they have no competing interests.

## Authors’ contributions

All authors contributed to the conception and design of the study; DR, PP and JD supervised the data collection; YC performed the statistical analysis and prepare the first draft of the manuscript. All authors contributed to the writing of the manuscript. All authors read and approved the final manuscript.

## Pre-publication history

The pre-publication history for this paper can be accessed here:

http://www.biomedcentral.com/1471-2466/12/32/prepub
